# Advances in human organs-on-chips and applications for drug screening and personalized medicine

**DOI:** 10.1016/j.fmre.2023.12.019

**Published:** 2024-02-22

**Authors:** Chenyang Zhou, Zhangjie Li, Kangyi Lu, Yijun Liu, Lian Xuan, Hongju Mao, Xiaolin Wang

**Affiliations:** a Department of Micro/Nano Electronics, School of Electronic Information and Electrical Engineering, Shanghai Jiao Tong University, Shanghai 200240, China; b Institute of Medical Robotics, Shanghai Jiao Tong University, Shanghai 200240, China; c State Key Laboratory of Transducer Technology, Shanghai Institute of Microsystem and Information Technology, Chinese Academy of Sciences, Shanghai 200050, China; d National Key Laboratory of Advanced Micro and Nano Manufacture Technology, Shanghai Jiao Tong University, Shanghai 200240, China; e National Center for Translational Medicine (Shanghai) SHU Branch, Shanghai University, Shanghai 200444, China

**Keywords:** Microfluidics, Organ-on-a-chip, Organoid, Drug screening, Personalized medicine

## Abstract

The limitations of conventional animal tests and two-dimensional cell culture hinder their advancement in fundamental research and clinical/translational applications. As an emerging alternative technology, organ-on-a-chip serves as a platform that faithfully simulates the key phenotypical, physiological, and functional features of human tissues/organs through the accurate regulation of the parameters such as physical and biochemical microenvironment, as well as cellular patterns. In this review, we mainly introduce the recent progress in the organ-on-a-chip field, including lung, gut, heart, liver, vasculature and multiorgan studies. Furthermore, we highlight the potential applications in drug screening and personalized medicine. Finally, we conclude the review by addressing the current challenges and future perspective in the technology and commercialization of organ-on-chips. We anticipate that the development of organ-on-a-chip technology will revolutionize the studies on biology and medicine by providing new understanding of mechanisms of diseases and insights into clinical therapeutics.

## 1 Introduction

The investigation of human physiology and pathophysiology is of great significance in the biomedical field, especially in understanding the disease mechanisms, drug development, clinical diagnosis and providing therapeutics. Currently, the methods predominantly rely on traditional animal models and two-dimensional (2D) cell culture systems, which suffer from corresponding challenges. The animal models not only are costly and time-consuming, but also raise ethical issues and have species differences. 2D cultures fail to accurately simulate the physiological-relevant structure, function, and the microenvironment cues of the tissues/organs. Consequently, these models lack validity and efficacy, and they are unable to accurately predict human responses, representing a major hurdle to efficient drug discovery and development [1]. Therefore, there is an urgent need for *in vitro* models that reproduce the critical structure and function of human organs and overcome the limitations of the conventional *in vitro* and *in vivo* models.

The organ-on-a-chip system is a technological integration of micro-manufacturing, tissue engineering and stem cell biology. It was selected as one of the “Top Ten Emerging Technologies of 2016” by the World Economic Forum. The goal of this system lies in the precise simulation of the physio/pathophysiological-relevant microenvironment of specific human tissues/organs through regulating key conditions such as the physical, chemical, and biological microenvironmental parameters, cell patterning, and tissues/organs interface. Therefore, with the principle of reductionist analysis of the target organ, the design concepts of organ-on-a-chip include cellular cues, mechanical cues, and biochemical cues. Human tissues/organs are complex and ordered arrangements of multiple cells that carry out their respective function. To support the 3D arrangement of target organs, microfluidic technology and biocompatible materials are required. Mechanical cues focus on two aspects: physiological-relevant stress/strain, such as alveolar relaxation process, intestinal peristalsis, and bone stress stimulation. Additionally, fluid shear forces or intercellular flow, such as blood/interstitial fluid flow, play a key role in cell differentiation physiologically [2]. Biochemical cues involve concentration gradients of molecules, including oxygen, growth factors, and drugs. The physiological 3D arrangements of organs cause concentration gradients, which in turn influence the occurrence of some processes, such as cell polarization [3] and angiogenesis [4].

Since the creation of the first organ-on-a-chip in 2010, which simulated the human breathing lung alveolus [5], a number of advanced organs-on-a-chip have been developed. These include models for the gut [6], liver [7], brain [8], blood–brain barrier [9], vasculature [10], heart [11], eyes [12], and bone [13]. A range of related diseases have been modeled *in vitro* based on organ-on-a-chip to reveal the mechanisms of disease and accelerate the development of new therapeutics screening. Combined with the organoids technology, organ-on-a-chip provides a promising platform to advance personalized medicine for individual patients’ differences [14]. By interconnecting different organ-on-chips through engineered blood vessels, multi-organ-on-chip or human body-on-a-chip can be constructed to study cross-organ interactions and pharmacokinetics (PK)/pharmacodynamics (PD) research [15]. Thanks to the collaborative efforts of academia and industry in the development of organ-on-a-chip technology, a legislation signed in late December 2022 states that new medicines no longer need to be tested on animals to receive the approval from the U.S. Food and Drug Administration (FDA) [16].

In this review, we first provide the concepts and prospects of organ-on-a-chip technology. We then introduce the recent advances in organ-on-a-chip technology, especially focusing on the lung, gut, liver, heart, vasculature, and the multi-organ-on-chip systems. Additionally, the applications of drug screening and personalized medicine are also summarized. Finally, we highlight the essential challenges and potential solutions in the field of organ-on-a-chip.

## 2 Critical organs-on-chips technology

### 2.1 Lung-on-a-chip

**Fig. 1:**
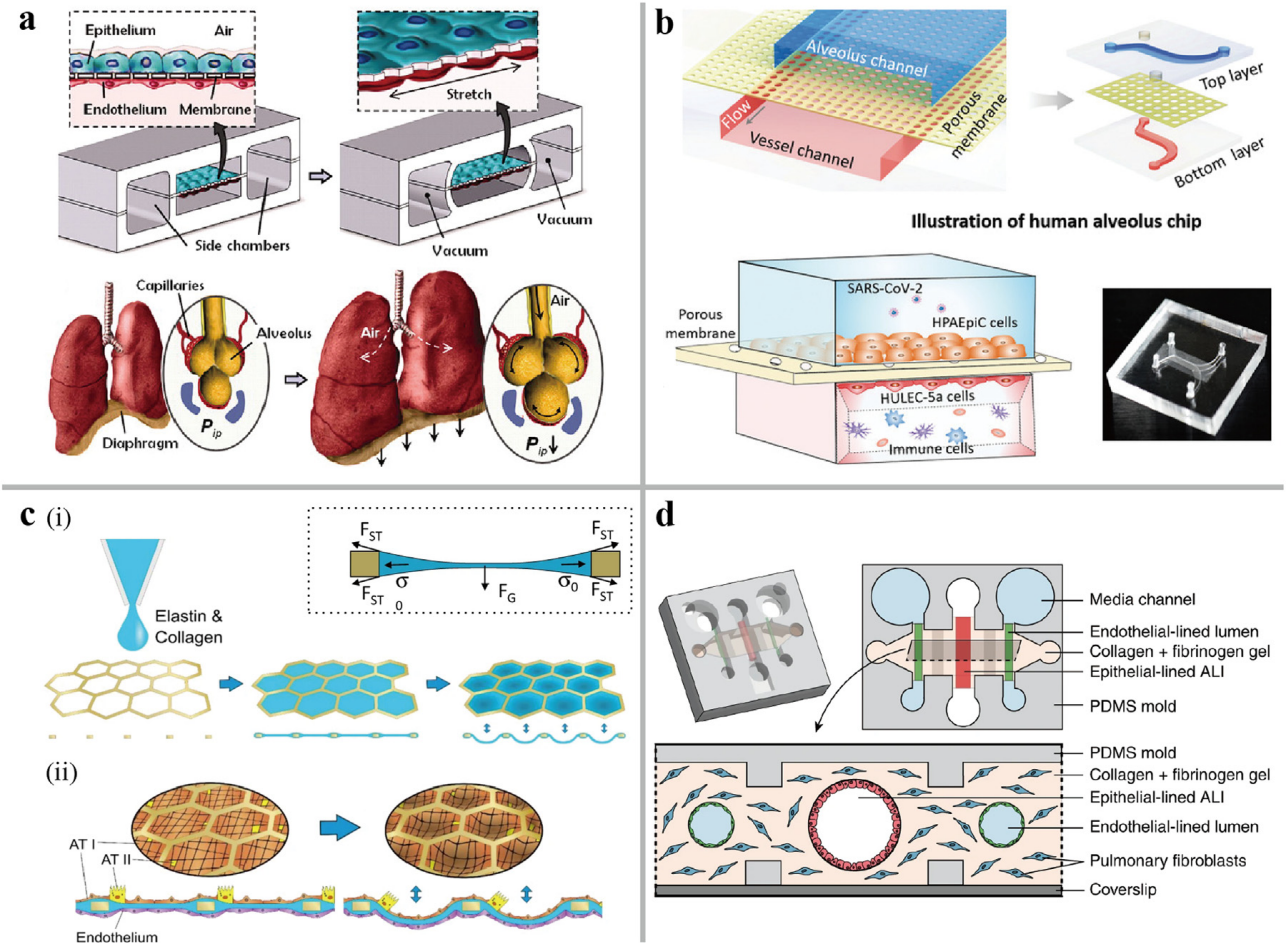
**The human lung-on-a-chip.** (a) Schematic of the lung-on-a-chip consisted of PDMS membrane coated with ECM to form alveolar-capillary barrier [5]. (b) Schematic of biomimetic human alveolus chip with the co-culture of SARS-CoV-2 and immune cells [21]. (c) Fabrication of a lung-on-a-chip with an array of stretchable alveoli made with a biological membrane [26]. (i) Production of biological membrane made of a thin gold mesh scaffold covered with the collagen-elastin (CE) hydrogel. (ii) Reconstruction of the stretchable alveoli on chip. Primary human lung alveolar epithelial cells are cocultured with lung ECs on the CE membrane that can be stretched at the alveolar level by applying a negative pressure on the basolateral side of the membrane. (d) Schematic of the organotypic bronchiole chip microfabricated on hydrogel [27].

As an organ of the human respiratory system, the lung has critical functions such as respiratory regulation and immunity. The lung consists of the conducting zone and respiratory zone. After flowing through the conducting airways, air exchanges between external environment and internal vessel systems occur through the continuous movement of the alveolar sac located in the bronchioles [17]. Microfluidic organ-on-a-chip systems have been developed to replicate the lung's blood-air barrier by implementing the dynamic perfusion and mechanical movement. In the pioneering work by Huh et al. [5], a lung-on-a-chip was designed in which two microchannels were separated by a porous Polydimethylsiloxane (PDMS) membrane, as shown in Fig. 1a. The human alveolar epithelial cells and microvascular endothelial cells (ECs) were seeded into the microchannels, which were coated with extracellular matrix (ECM). Cyclical mechanical stretching of the flexible PDMS membrane using a vacuum pump simulated the breathing movements. Later, Interleukin-2 (IL-2) and angiopoietin-1 (Ang-1) were sequentially introduced into this model to replicate the pathophysiology of pulmonary edema [18]. This sandwich structure is well appropriate for respiratory modeling. More importantly, one of the most clinically relevant applications of lung-on-a-chip is in respiration research [19]. For example, a cystic fibrosis airway-on-a-chip using primary epithelial bronchial cells isolated from patients successfully reproduced the characteristics of respiratory dysfunction observed clinically: increased ciliary activity, mucus accumulation, and enhanced colonization of *P. aeruginosa* in the mucus [20]. Another human lung-on-a-chip, co-culturing severe acute respiratory syndrome coronavirus 2 (SARS-CoV-2) and circulating immune cells, revealed that recruitment of circulating immune cells exacerbates lung inflammation, endothelial dysfunction, and injury of lung barrier (Fig. 1b) [21]. Infection of a lung airway-on-a-chip lined by human lung microvascular ECs (HMVEC-L) and normal human bronchial epithelial (NHBE) cells suggested that the decrease of Claudin-5 (CLDN5) expression caused by SARS-CoV-2 leads to the disruption of vascular barrier in the lung and fluvastatin is a potential therapeutic drug to upregulate the CLDN5 expression [22]. When IL-13 was used to treat on a small airway-on-a-chip in another study, it was observed that the higher levels of the inflammatory cytokines and a decrease in cilia beating frequency, which were characteristics of asthma [23]. Additionally, chronic obstructive pulmonary disease (COPD) epithelial cells were seeded on this small airway-on-a-chip to analyze the COPD exacerbation caused by pathogenic infections. The above models demonstrate the robust functions of the sandwich-structure chip in disease modeling, drug trails, and personalized medicine. However, the structure and function of organs/tissues are intrinsically connected, as evidenced by the significance of topography/topology in determining cell fate [24]. In a study that took into account the microscale curved surfaces of alveoli, the membranes with the shape of hemispherical microwells were fabricated by a combination of the microthermoforming and ion track technology [25]. The Calu-3 lung epithelial cell line and HMVEC-L were seeded on the two sides of the membranes, and this model enables reconstructing spatial configuration of the alveolar sac.

Though PDMS has well optical transparency, elasticity and biocompatibility, its high adsorption and absorption level of small molecules on PDMS interface interfere with controlling microenvironment in chip [28]. On the other hand, this non-natural substrate lined with cells impacts the cellular phenotype and homeostasis [29,30]. In this case, the hydrogel material has received substantial attention. As shown in Fig. 1c, a biological membrane with good transparency, biodegradability, tensile strength, and low adsorption level to rhodamine-B was created by drop-casting a collagen-elastin solution onto a gold mesh scaffold [26]. This chip supported the co-culture of the alveolar epithelial cells and ECs, which presented more physiologically relevant phenotypes and adhesion compared to culturing on PDMS membrane. Furthermore, the borders of the alcoves mimicking the alveolar walls produced non-uniform mechanical strain spatially, which is consistent with the complex mechanical strain conditions under different thickness and complex parenchymal structure of alveoli [31]. Barkal et al. designed a novel organotypic bronchiole chip by ECs-lining collagen hydrogel lumens embedded with fibroblasts (Fig. 1d) [27]. This biomimetic system replicated the complex structure of human bronchiolar endings, demonstrating the volatile interaction between organotypic bronchiole model and *Aspergillus fumigatus*.

### 2.2 Gut-on-a-chip

**Fig. 2:**
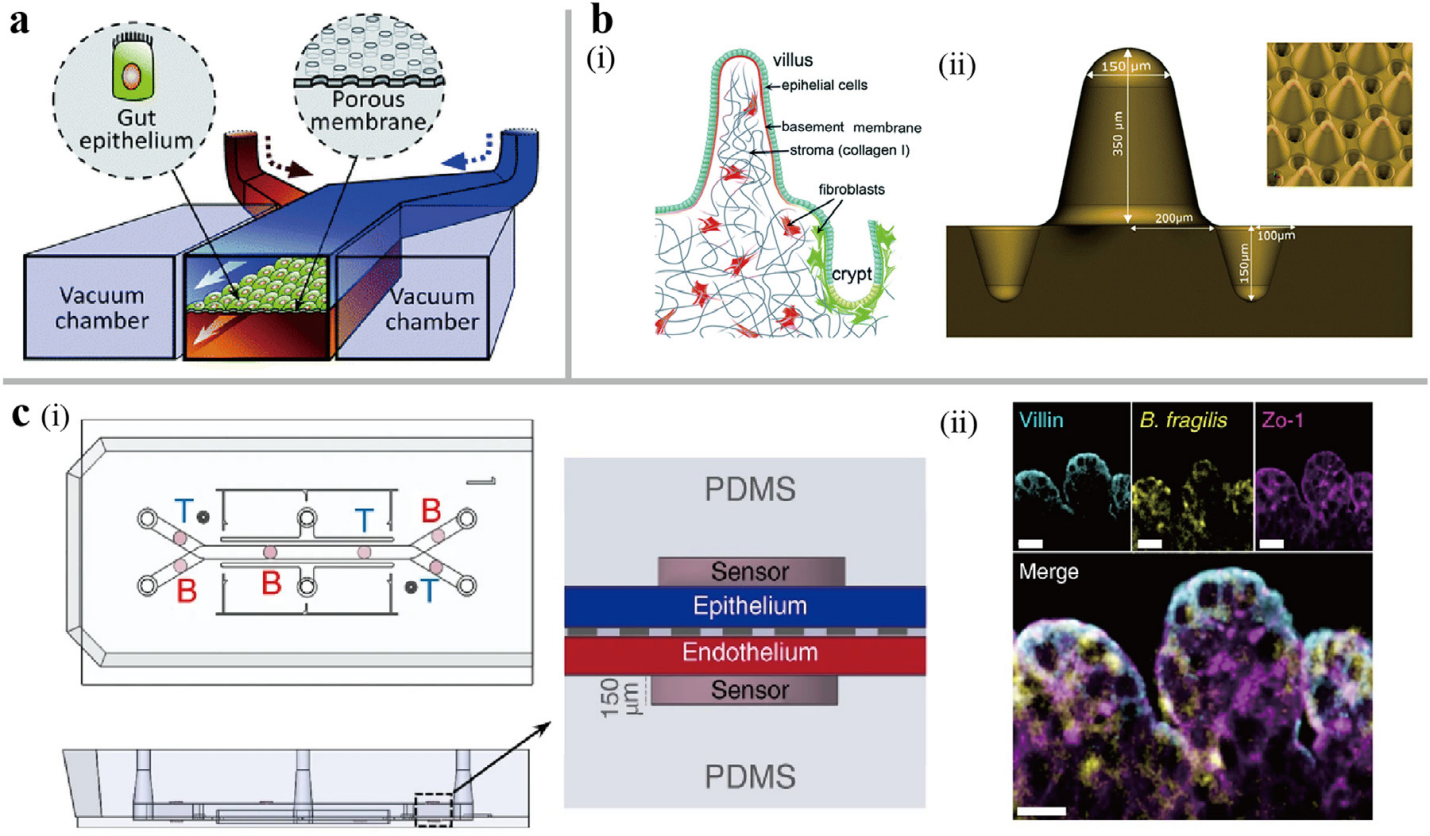
**The human gut-on-a-chip.** (a) Schematic of a gut barrier-on-a-chip device. The flexible porous membrane divides two flow chambers, which is lined by gut epithelial cells and ECs, respectively. The vacuum chambers are utilized to simulate the intestinal peristalsis [6]. (b) Schematic of a device that constructs 3D collagen scaffold mixed stromal cells with the typical surface topography of the mouse gut. (i) Schematic of the small intestine. (ii) The contour brass mold is used to model the PDMS stamp. Villus height 350 µm, crypt depth 150 µm [37]. (c) The anaerobic gut-on-a-chip system integrated oxygen sensors to study the gut cells-microbiome interactions. (i) Schematic of the microfluidic chip with six oxygen-quenched fluorescent particles. (ii) Representative confocal image through the intestinal epithelium−microbiome interface under anaerobic conditions. Villin, ZO-1 and B. fragilis were stained and appeared in cyan, magenta and yellow, respectively. Scale bar, 50 µm [40].

The human gut is an important organ responsible for food digestion, absorption and immune process, whose motility maintains the bodily functions. The unique crypts and villi structures are fundamental, self-renewing, and functional structure units in the gut [32]. The large-surface villi and periodic peristalsis promote the digestion and absorption in the small intestine. The villi provide suitable niches for diverse cell populations and microbial communities in the intestinal epithelium [33]. A two-channel, mechanically actuated organ-on-a-chip similar to sandwich-structure lung chip can be utilized to recapitulate the gut barrier function (Fig. 2a) [6]. In addition, the simulation of the gut microenvironment, including the intestinal peristalsis and physiological fluid, promoted the polarization of the columnar epithelium that spontaneously grew into folded intestinal villi structures [34]. Moreover, the complex gut structure can be modeled using a hydrogel matrix scaffold with higher biocompatibility. A gut-on-a-chip was developed by culturing Caco-2 cells adhered to the ductal scaffold of collagen hydrogel created by needles [35]. The compressive stress arising from the cell proliferation in the limited ductal scaffold area induced the epithelial fold structure formation without the activation of peristalsis. After 16 days of the culture period, a thicker intestinal epithelial barrier than a gut monolayer model formed, suggesting that epithelial cells differentiated from squamous cells to columnar cells. At the cellular level, the ductal scaffold translated Caco-2 cells into apically polarized cells, as evidenced by an increased epithelium height, a columnar cell-like aspect ratio, and vertical villi lining. Hydrogel scaffold mimicking topography of gut crypt-villi architecture could be achieved through engineering approaches. Wang et al. designed a PDMS stamp by soft-lithography method, and pressed it into a collagen hydrogel solidified on a Transwell membrane [36]. Cells cultivated on the scaffold were induced to form a crypt-villi structure and polarized through a gradient of soluble biochemical factors. Furthermore, the hydrogel allows the co-culture of primary epithelial and stromal cells [37]. This gut-on-a-chip was created by culturing epithelial cells on fibroblasts-mixed collagen scaffold with physiological topography and dimensions of the crypt-villi. A layer of laminin was coated on the hydrogel surface, simulating the basement membrane of gut (Fig. 2b). The model recreated the epithelial-stromal cells interactions, allowing for the research of the human gut physiology and pathophysiology.

The human gut is also the host of a variety of microbiota, which is vital for maintaining the intestinal homeostasis, nutrient absorption, and drug metabolism. The gut harbors approximately 10^14^ microbial cells, predominantly consisting of anaerobic bacteria. Consequently, a physiological oxygen gradient is another distinguishing characteristic of the human gut [38]. Measurements indicate the presence of a vertical oxygen gradient stretching from the vascularized submucosa across the villus tip [39]. To recreate this oxygen gradient *in vitro*, a gut-on-a-chip was developed in which the host-microbiome channel was flushed with a mixture of 5% CO_2_ and N_2_ gas to maintain a low oxygen level. The oxygen gradient was achieved through the oxygen diffusion from the permeable PDMS membrane in the endothelium channel (Fig. 2c) [40]. This chip supported real-time and non-invasive measurements via oxygen-quenched fluorescent sensors. The co-culture of epithelium and bacteria in physiologically relevant oxygen conditions significantly enhanced the barrier function. Another study reported a way that N_2_ gas was constantly bubbled into the medium until the concentration of oxygen in the medium was below 0.1% prior to perfusion [41]. Research on host-microbial interactions on this anoxic gut-on-a-chip showed that the co-culture changed the expression of miRNAs related to colorectal cancer in Caco-2 cells, and led to the accumulation of GABA (4-aminobutanoic acid) in epithelial cells induced by *Lactobacillus rhamnosus GG*.

The integrity of the gut barrier is a key to the absorption and transport of small molecules [42]. To measure the barrier function, the perfusion of molecules with high molecular weight such as fluorescein isothiocyanate dextran and rhodamine-labeled dextran can be used [43]. Immunofluorescence staining of proteins involved in various junctional complexes responsible for permeability and barrier integrity is another assessment method. Commonly, the junctional complex proteins include the epithelial (E)-cadherin for epithelial tight junctions, the vascular endothelial (VE)-cadherin for endothelial adherens junctions [44] and the zonula occluden-1 (ZO-1) proteins for epithelial-endothelial junctions [45]. Based on the cell monolayer model comprising a capacitor and a resistor, the trans-epithelial/endothelial electrical resistance (TEER) measures the electrical resistance to characterize the barrier integrity, where a high TEER value indicates tight junction formation [46]. Liu et al. utilized TEER to assess the leakage of epithelial tight junctions following exposure to a cocktail mixing with tumor necrosis factor-α (TNF-α) and lipopolysaccharides (LPS) on the gut-on-a-chip [47]. Reliable data can be obtained from large-scale TEER that generates the homogeneous electric field, but the architecture made of Au/Pt interferes with the microscope observation. A semitransparent electrode made of organic semiconductor polymer poly (3,4-ethylenedioxythiophene) doped with polystyrene sulfonate (PEDOT:PSS) was integrated into the gut-on-a-chip, enabling real-time monitoring of the gut barrier development and the changes in barrier function upon exposure to a permeability enhancer [48].

### 2.3 Heart-on-a-chip

**Fig. 3:**
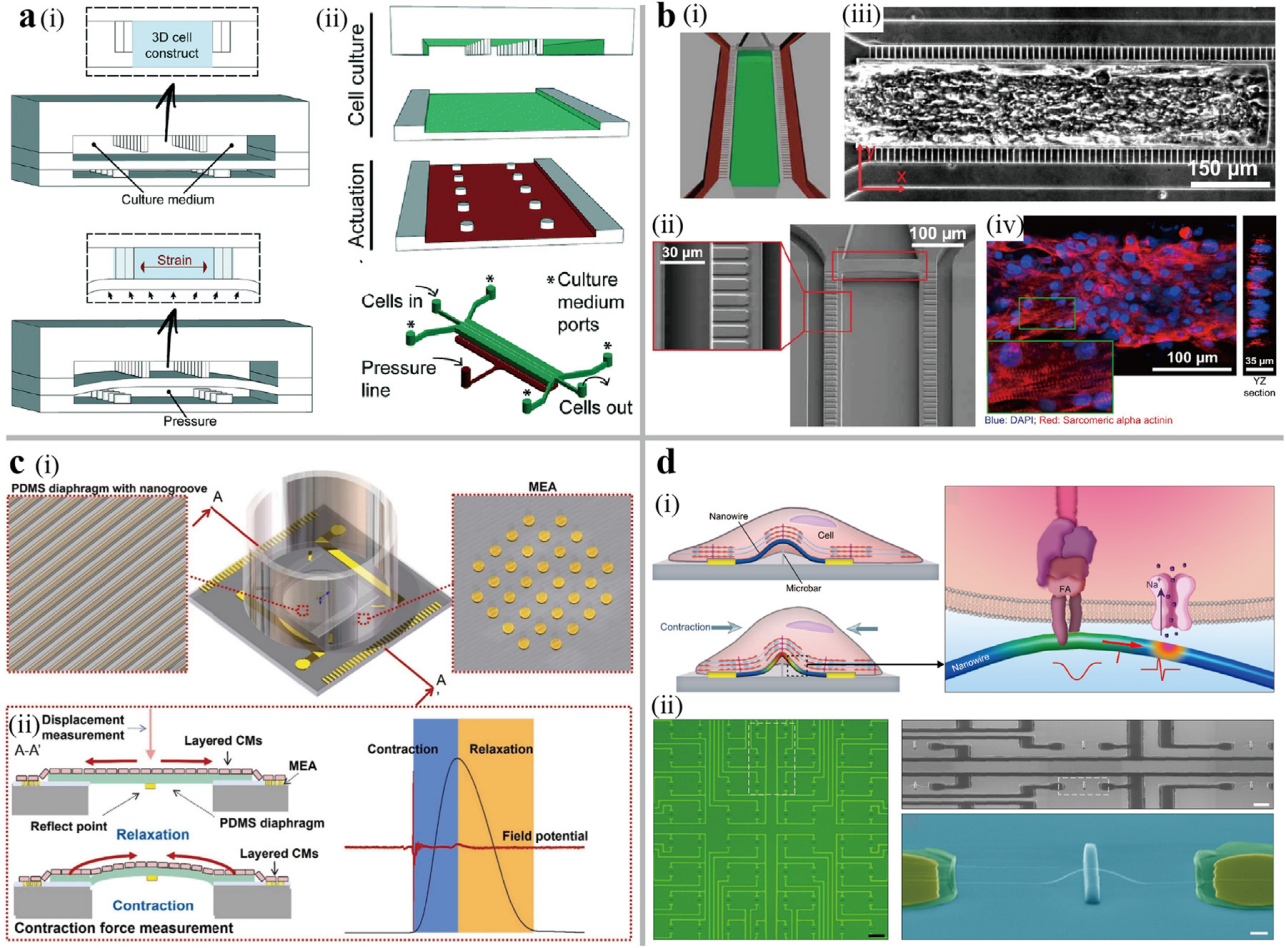
**The human heart-on-a-chip.** (a) Schematic of a 3D heart-on-a-chip. (i) The diagram of the working process of the uniaxial strain stimulation. (ii) The schematic of the fabrication process, where the three PDMS layers were aligned and bonded [53]. (b) A heart-on-a-chip using iPSC—CMs. (i) the diagram of the cell chamber (green) and medium channels (red). (ii) SEM image of the 2 µm microchannels connecting cell chamber and medium channels. (iii) The image of the cardiac tissue in the heart-on-a-chip. Scale bar, 150 µm (iv) Confocal fluorescence microscopy image of the cardiac tissue, where the sarcomeric alpha actinin is labeled red and cellular nure is labeled blue [56]. Scale bar, 100 µm (c) (i) Schematic of a biosensing platform for simultaneous measurements of contraction force and field potential of cardiomyocytes. (ii) The sensing principle of mechanical and electrical information measurement, and the correlation between the signal and time [61]. (d) Schematic of a nanotransistor sensor for electrical and mechanical responses of cardiac tissue [62]. (i) The electrical coupling and mechanical coupling between the nanowire and cell for the simultaneous detection of cellular force and active potential. (ii) The optical image and scanning electron microscope image of the device.

The heart is a unique organ that diseases such myocardial necrosis and heart failure can lead to an irreversible damage. Currently, the heart transplantation is the only clinical treatment available for such diseases. Therefore, heart-on-a-chip is a promising approach for research on cardiac biological mechanism and regenerative medicine. The mature cardiomyocytes are cylindrical in shape with an aspect ratio of 7:1, which can be reconstructed on heart-on-a-chip through microscopic structural design [49]. Morris et al. developed a model by patterning fibronectin lines with 20 µm width and 5 µm gap on a PDMS substrate using microcontact printing [50]. They cultured cardiomyocytes and skeletal muscle on this chip and evaluated the contraction intensity by quantifying α-actinin skeleton and orientation via image processing. This cardiac model cultured on fibronectin lines produced a stronger contractile force when compared with those on non-patterned substrates. Additionally, continuous electromechanical loading on the heart-on-a-chip supported the structural, functional, and transcriptional changes of myocardial slices [51,52]. It was found that the replication of physiologically relevant electromechanical stimulation improved and changed the myocardial structure, function, and transcriptional level *in vitro*. To simulate the systolic and diastolic phases of the cardiac tissue, a chip was designed that the culture chamber and air chamber were divided by a PDMS membrane (Fig. 3a) [53]. Applying a pressurization, the membrane moved upwards to induce a uniaxial strain stimulation of the cells in the upper culture layer. The study observed that the mechanical stimulation accelerated the cardiac maturation and enhanced the spontaneous beating and contractility. To further study the dynamic mechanical changes of heart *in vivo*, Fotios et al. developed an electro-mechanical system that accurately simulated mechanical loading based on physiologically relevant parameters [54]. It was found that the contractility of rat living myocardial slices decreased under the excessive loading, and the PI3K-Akt pathway was dysregulated in this process. Miller et al. simplified the entire system to allow for long-term culture, medium throughput and low cost, which shows the potential for cardiac preclinical drug discovery [55].

Tissue biopsies and primary cells are the main cell sources for the above heart-on-a-chip models, but their widespread availability is limited by culture times and changes in cellular phenotype. One solution to this problem is the use of cardiomyocytes, which can be differentiated from human embryonic stem cells (hESC—CMs) or pluripotent stem cells (iPSC—CMs) through chemical, physical and biological stimulation. Mathur et al. developed a heart-on-a-chip using iPSC—CMs, as shown in Fig. 3b [56]. The cell chamber was connected to the medium channel by 2 µm wide microchannels, which not only blocked the cells leakage from chamber to channel, but also prevented the cells from shear forces. In another study of heart-on-a-chip by Kujala et al., human iPSC—CMs were cultured on the multi-electrode array (MEA) coated with micromolded gelatin [57]. The laminar cardiac tissue induced from iPSC—CMs through the topographical cues facilitated the assessment of the cardiotoxic pro-drug terfenadine and its non-cardiotoxic metabolite fexofenadine. Another strategy involves the integration of 3D bioprinting technology and microfluidic technologies to recreate endothelialized myocardial tissue from iPSC—CMs, which was utilized to evaluate the responses of heart myocardium to the anti-cancer drug doxorubicin [58].

The contractile function is an integral parameter for research on cardiac physiology and responses to drug interventions using heart-on-a-chip models. In this case, it is necessary to integrate biosensing components to dynamically monitor the heart-on-a-chip [59]. The primary parameters used for evaluating contractile functions in cardiac models include the beating frequency, action potential and contraction force. Furthermore, a multiparameter analysis of cardiac function is vital to elucidate the mechanism of contraction or drug discovery, as it provides detailed time-dependent relationships. A triple-parametric optical mapping system consisting of 450 nm, 590 nm, and 690 nm filters, along with CMOS cameras, simultaneously measured the spectral intensity to quantify NADH, Ca^2+^, and transmembrane potential signals, respectively [60]. The target effects of blebbistatin, 4-aminopyridine and verapamil on cardiac physiology were tested by the metabolism-excitation-contraction (MCC) methodology. Zhao et al. fabricated a heart-on-a-chip where the laser vibrometer measured the displacement of the PDMS substrate to reflect the contraction force of the cardiomyocytes (Fig. 3c). Additionally, the MEA integrated into the PDMS membrane measured the field potentials of the cardiomyocytes [61]. Furthermore, a nanotransistor sensor can concurrently measure electrical and mechanical responses of cardiac tissue (Fig. 3d) [62]. In this design, the deformation or strain change in the nanowire, which results from contraction of cardiac tissue, can generate signals by the piezoresistive effect, while the nanotransistor can also detect the action potentials through the field effect. This heart-on-a-chip model was used to recapitulate the different mechanisms of the cardiovascular drug blebbistatin, lidocaine, and isradipine.

### 2.4 Liver-on-a-chip

**Fig. 4:**
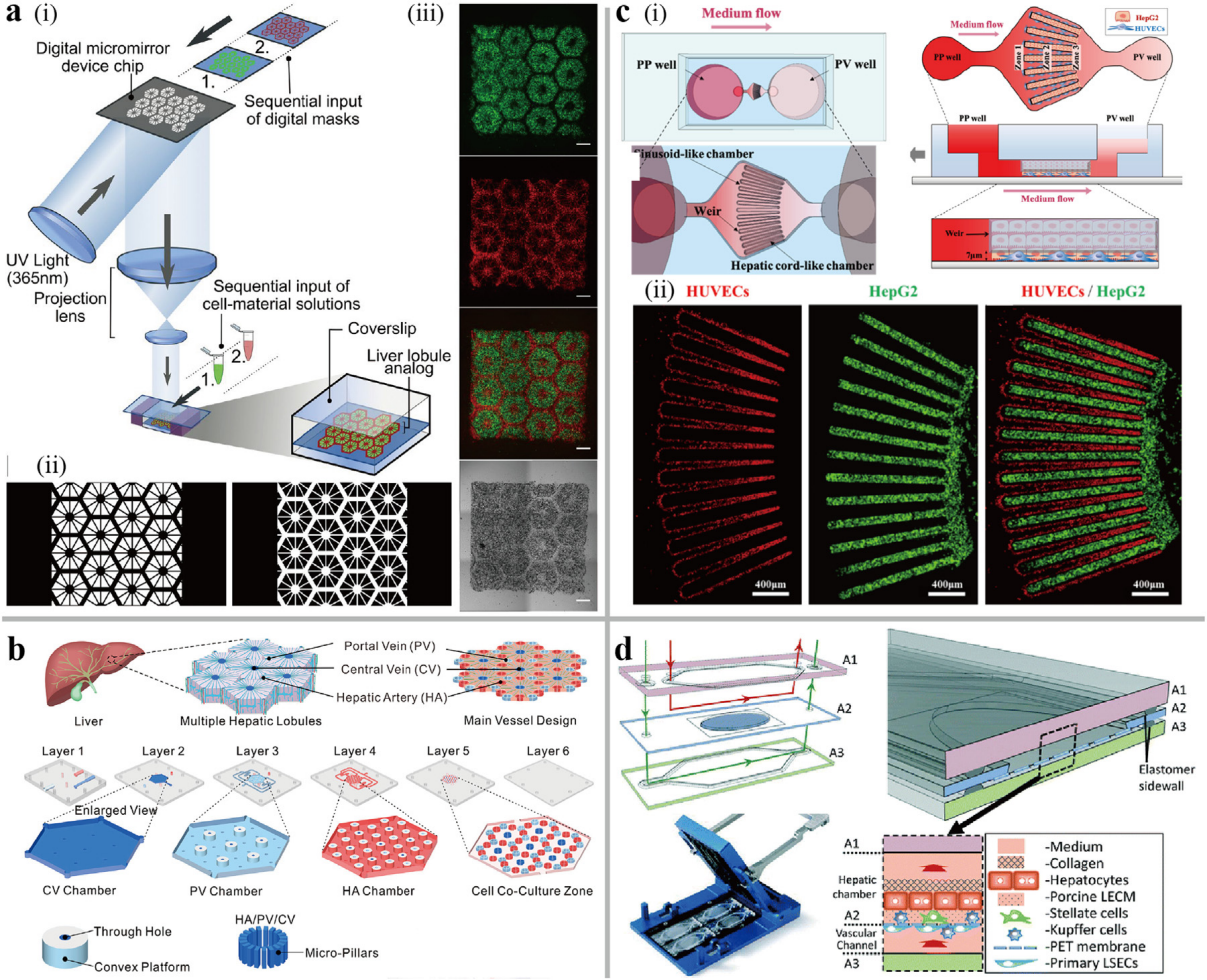
**The human liver-on-a-chip.** (a) Schematic of DLP-based 3D bioprinting hepatic tissue. (i) Fabrication of two-step 3D bioprinting process; (ii) the masks of liver lobule and vascular structure, respectively; (iii) The fluorescent and bright field images of liver lobule model, hiPSC—HPCs and stromal cells were stained and appeared in green and red [64]. (b) Schematic components of engineered liver lobule chip with PVs, HAs, and CVs [65]. (c) Schematic of the liver acinus chip. (i) the configuration of the chip wit alternate chambers in a radial shape to seed HepG2 and HUVECs; (ii) The fluorescent images of liver acinus radial pattern [67]. Scale bar, 400 µm. (d) The configuration of a vascularized liver acinus chip [68].

Liver is the largest internal organ in the human body, weighting about 1.4 kg (2.5% of an adult weight), and plays a crucial role in drug metabolism, the protein production, and the regulating glucose and lipid titers level [63]. Therefore, it is imperative to develop biomimetic liver-on-a-chip models for *in vitro* physiology or pathophysiology studies and drug discovery. Ma et al. utilized digital light processing (DLP)-based 3D bioprinting to design a liver model that recreated the complex structure of liver lobules and interaction between hiPSC-derived hepatic progenitor cells (hiPSC—HPCs) and stromal cells (Fig. 4a) [64]. Following the tri-culture, the cells reorganized in their designated patterns, and the functions of hiPSC—HPCs were enhanced, including higher liver-specific gene expression and increased metabolic product secretion. Inspired by lattice growth mechanisms in material science, a liver lobule-on-a-chip with a hexagonal culture chamber was designed to mimic an organotypic hierarchy [7]. The culture chamber, made of a collagen-coated PDMS membrane, was enclosed with a hydrophilic flow diverter and seeded with primary liver cells. The medium, which was powered by peristaltic pump, flowed from six inlets at each corner of the hexagonal culture chamber to the outlet in the center of the chamber, thereby simulating the flow from the portal vein to the central vein. This chip reconstituted the ability to reconstruct hierarchical tissue without the aid of ECM scaffold. Ya et al. designed and fabricated a liver chip consisting of six layers with different structures and functions (Fig. 4b) to simulate the physiological flow of portal veins, hepatic arteries, and central veins in the liver lobular [65]. Furthermore, an oxygen concentration regulating chip was connected before the inlets of portal veins and hepatic arteries to provide physiologically precise concentrations of dissolved oxygen in dual vessels. Self-assembled perfusable hepatic sinusoids formed between the liver lobules under the flow at a certain velocity. Their results also showed that differentiated oxygen concentrations contribute to the long-term, large-scale hepatic tissue formation.

Metabolically, the hepatic acinus serves as the functional unit of the liver, divided into three zones: Zone 1 periportal (PP), Zone 2 transitional, and Zone 3 perivenous (PV) [66]. To mimic the structure of the liver acinus and hepatic zonation, HepG2 cells and HUVECs were alternatingly patterned as a partial hexagonal shape (Fig. 4c) [67]. The oxygen/glucose-containing medium flow contributed to create the metabolic zonation, which was proved by the respective expression of periportal and perivenous markers. Another liver acinus model was designed by culturing multiple human liver cell types in a glass-based chip with two microchannels separated by polyethylene terephthalate (PET) membrane [68]. As depicted in Fig. 4d, ECs and Kupffer cells were seeded at the bottom of membrane with the aid of fluidic stimulation. A liver extracellular matrix (LECM) solution was introduced, and hepatic cells were cultured on a LECM-coated layer that simulated the space of Disse (perisinusoidal space). In addition, the cell types and seeding numbers were guided by allometric scaling. Continuous oxygen zonation was achieved by controlling the flow rates through the oxygen impermeable materials, which replicated the physiological zone-dependent metabolic functions of human liver *in vivo*.

### 2.5 Vasculature-on-a-chip

**Fig. 5:**
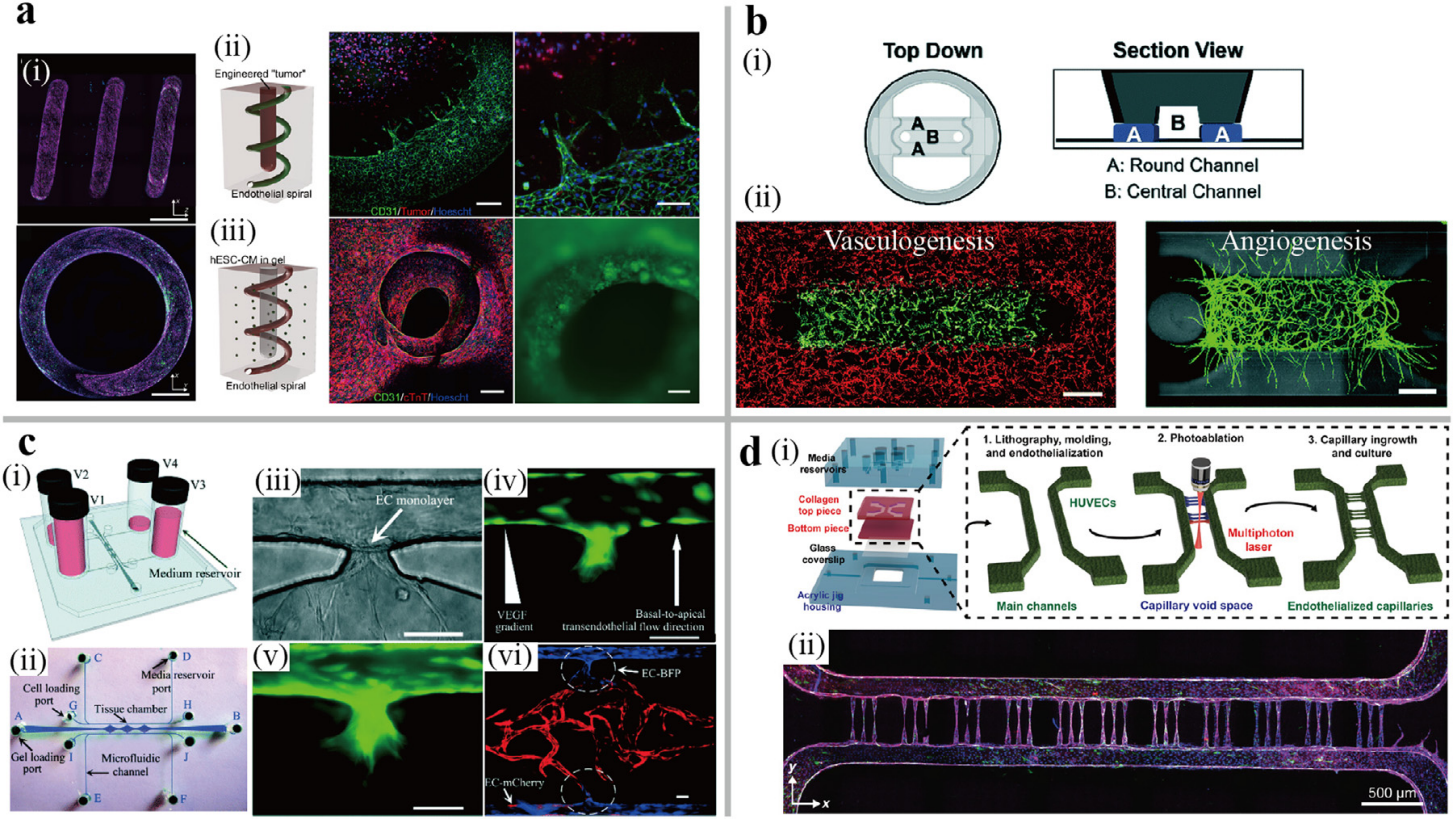
**The human vasculature-on-a-chip.** (a) A spiral microvessel system with robust endothelium along the lumen [72]. (i) Optical section of the spiral vessel. (ii) Engineered vascularized tumor model with the sprouting vascular toward tumor cells. Scale bar, 200 µm. (iii) Vascularized cardiac model. Scale bar, 500 µm. (left) and its effect can be studied in endothelialized spiral vessels (b) The “Open-top” vasculature-on-a-chip supporting various configuration of hydrogel patterning for vasculogeneis and angiogenesis [79]. (i) Schematic of the chip in a top and section view. (ii) confocal images of vasculature in the process of vasculogenesis and angiogenesis, respectively. Scale bar, 500 µm. (i) Schematic of the device in which the HUVECs and NHLF were separated in two defined regions. (ii) Fluorescent image of the formation of open lumens in which the ECs invade to the other side. Scale bar, 100 µm. (c) The vasculature combined vasculogenesis and cell-lining [10]. (i) and (ii) Schematic of the whole microfluidic device and the top-view of channel structure. (iii)-(vi) Sprouting vasculogenesis and anastomosis. Scale bar, 100 µm (d) The capillary-sized vasculature formed by photolithography and femtosecond laser photoablation [80]. (i) Schematic of the vasculature-on-a-chip assembly and capillary fabrication. (ii) confocal fluorescent image with complete vessel network consisting of 33 capillaries. Scale bar, 500 µm.

Vasculature is a ubiquitous tissue in human body responsible for transporting nutrients and waste, maintaining homeostasis, and shaping tissue/organ structure [69]. Strategies for fabricating 3D vascular networks can broadly fall into two categories: the top-down engineered vasculature and the bottom-up self-assembled vasculature. The top-down approach constructs engineered microchannels lined with ECs to achieve controlled geometry and dimensions of the microvasculature. PDMS and hydrogel are two main materials used for the microchannel walls. However, PDMS has a higher modulus than that of physiological vasculature, damaging the endothelial barrier function [70]. To construct a high-fidelity microvascular network with physiologically relevant stiffness, the agarose-gelatin interpenetrating-polymer-network hydrogel was casted on the master mold using the soft-photolithography method [71]. This model recreated the responses to barrier-disrupting inflammatory mediators and the pathologies of sickle-cell disease and malaria while maintaining the endothelial barrier function for up to one month. Mandrycky et al. developed a spiral vessel by polymerizing the hydrogel around a spring, resulting in an additional central lumen for independent access and cell seeding (Fig. 5a) [72]. The increased flow in spiral geometry was found to enhance endothelial cells alignment in the direction of flow and promote the proliferation, which was contrary to those cultured in straight lumen. Considering the vascular network *in vivo*, this model can contribute to the research of differential development and pathogenesis caused by heterogeneous vascular structures. By combining the biocompatible polymer microchannels with traditional plastics consumables 96-well plates that avoid the drug absorption, the microfluidic vasculature-on-a-chip allowed quick translation into biosafety level-3 laboratories [73]. SARS-CoV-2 was introduced to study the vascular dysfunction led by the inflammatory reaction of monocytes and macrophages. Moreover, QHREDGS peptide has shown its potential as the anti-SARS-CoV-2 therapeutic, reducing the release of the endothelial pro-inflammatory factors to inhibit damage of the endothelial barrier function. However, the EC-lined approach above cannot describe the vascular formation process *in vivo*, which mainly relies on the natural process of vasculogenesis and angiogenesis to form the complex microvascular networks [74].

The bottom-up self-assembled vascular network is a more physiologically relevant process that mimics the invasion from pre-existing vessels into a hydrogel in response to an angiogenic gradient analogous to angiogenesis, or the process of de novo spontaneous vascular formation analogous to vasculogenesis. A perfusable vascular network model was developed in which co-culture spheroids were used to mimic the interaction between the ECs and fibroblasts under the angiogenesis mechanism [75]. It was observed that moderate levels of adhesion, appropriate concentration of growth factor, and frequency of medium exchange were integral for angiogenesis. The co-culture spheroids, consisting of normal human lung fibroblast (NHLF) and human umbilical vein endothelial cells (HUVECs), were anastomosed with the angiogenic vascular network, which supported the perfusion and the endothelial barrier function. To improve the maturity of the vessels in the process of vasculogenesis, a fibrin hydrogel embedded with NHLF and HUVECs was introduced into the microfluidic vasculature-on-a-chip to form a perfusable vascular network under the stimulation of fluidic shear stress and growth factors [76]. This vasculature-on-a-chip was standardized to fit into a 96-well plate format, allowing for the large-scale drug screening applications. *In vivo*, fibroblasts become activated to release essential growth factors and chemokines in conditions such as wounding. Thus, fibroblasts contribute to regulating and maintaining the ECM homeostasis, and improving the formation of vascular lumens. However, this direct contact between ECs and fibroblasts on the chip differs from the cell-cell communication that occurs *in vivo*. In this case, a microfluidic vasculature-on-a-chip was designed as a non-contact cell culture arrangement to investigate the paracrine communication of the co-culture. In this arrangement, the hydrogel channels of each cell type were divided by the medium channel [77]. The analysis identified that versican (VCAN), intercellular adhesion molecule 2 (ICAM2) and tenascin C (TNC) have the potential to promote the maturation of vascular networks.

One single microfluidic device can model two different mechanisms of the bottom-up self-assembled vasculature through the control of the cell patterning and microenvironment on chip. Oh et al. reported a five-channels microfluidic chip to simulate vasculogenesis and angiogenesis [78]. The device utilized a blend of NHLF and human dermal microvascular endothelial cells (HDMEC) in a fibrin hydrogel for vasculogenesis and introduced ECs into the medium channel to adhere on the fibrin hydrogel mixed with fibroblasts for angiogenesis. An “Open-top” microfluidic chip also allowed for an agile cell patterning on different regions to replicate vasculogenesis, angiogenesis, and anastomosis [79]. As shown in Fig. 5b, round channel and center channel can support the hydrogel patterning and the subsequent vasculogenesis. Additionally, the anastomosis between the RFP- (center patterning) and GRP HUVECs (round patterning) was visualized for the investigation of cellular interaction through direct contact. For the process of angiogenesis, hydrogel mixed with HUVECs and LF was introduced into the center channel while loading acellular hydrogel into the round channel. It was found that higher concentration of LF resulted in longer sprouting vessels in the round region. Wang et al. purposed a vasculature-on-a-chip design that combined cell-lining and vasculogenesis to recreate the artery-vein-capillary systems (Fig. 5c) [10]. The vasculogenic vessels in the diamond chamber anastomosed with the cell-lining vessels in medium channels. The fluorescein isothiocyanate (FITC)-dextran perfusion demonstrated non-physiologic leakage. Further, multiphoton microscopy-guided femtosecond laser photoablation was used to generate the engineered vessels physiologically relevant to the capillaries *in vivo* (Fig. 5d) [80]. First, the two main microchannels were lithographically fabricated to simulate the large arteriole or venule in collagen hydrogel. Then, the femtosecond laser photoablation generated the capillary-sized microchannels, and incurred local ECs injury for successful enothelialization. It was observed that malaria-infected red blood cells (RBCs) accumulated in the narrow capillary regions, while the normal RBCs readily traversed. This vasculature model represents an improvement in accurately recapitulating physio/pathophysiological vascular structures *in vitro*.

### 2.6 Multi-organ-on-chip system

Numerous Organ-on-a-chip models recapitulating virtually all tissues/organs and their interfaces in human body have been proposed in the past decade. However, the complex physiological or pathophysiological processes and responses *in vivo* rely on cross-organ communication mediated via circulating various signals in blood and lymph [81]. For instance, the oral medications require sequential events called ADMET that involve various organs: absorption (gut), distribution (blood circulation), metabolism (liver), excretion (kidney), and toxicity (a target organ) [82]. Another example is that metastasis of tumor cells and colonization into the distant organs (brain, bone, liver, etc.) driven by circulating tumor cells (CTC) is the leading cause of cancer mortality [83]. Thus, it is necessary to develop various multi-organ-on-chip system containing different interconnected representative tissues.

**Fig. 6:**
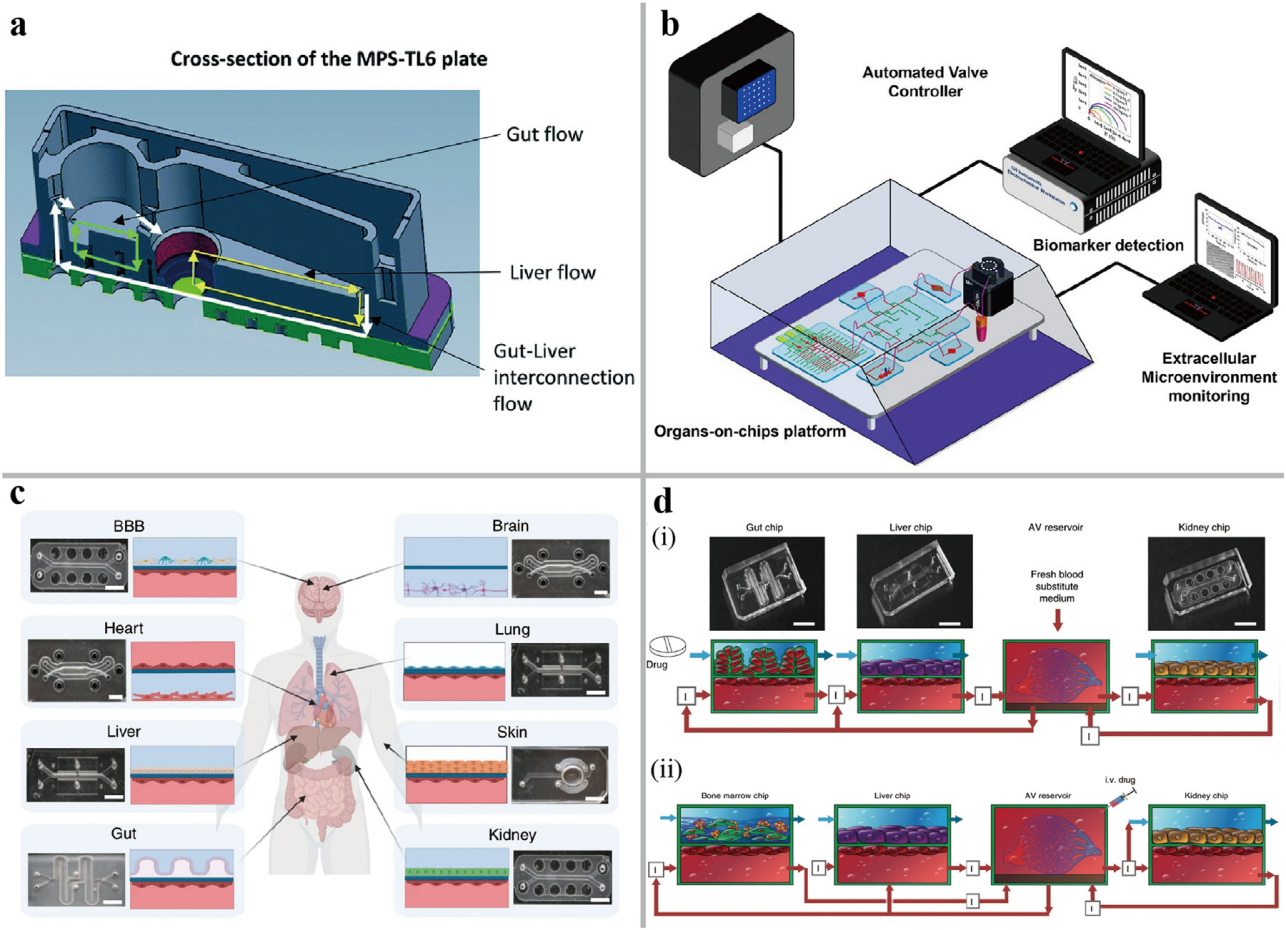
**The multi-organ-on-chip systems.** (a) Schematic of a gut-liver-on-chip system [85]. (b) A multisensors-integrated multi-organ-on-chip system, consisting of automated valve controller, biomarker detection, extracellular microenvironment monitoring and organs-on-chip platform [86]. (c) The representative images and sectional schematic of a multi-organ-on-chip system consisting of brain–blood-barrier (BBB), heart, liver, gut, brain, lung, skin, and kidney [87]. (d) physiological PK modelling of first-pass drug absorption, metabolism and excretion [88]. (i) The model coupling gut, liver and kidney chips for simulation of orally administered nicotine. (ii) The model coupling bone marrow, liver and kidney chips for simulation of intravenously injected cisplatin.

Multi-organ-on-chip can fall into two types by engineering methods of fluidic coupling: integrated plates and modular units. Integrated and compact multi-organ-on-chip plates have fewer detachable components for limiting the fluid leakage, while the modular units connected via capillary tubing at any given time allow redundancy capacity and flexible configuration [84]. A gut-liver-on-a-chip was designed consisting of a gut compartment with a Transwell to mimic small intestine barrier function, and a liver compartment under the simulation of shear stress for primary hepatocytes (Fig. 6a) [85]. Powered by a system of pumps, there were three individually controlled fluids to support gut-liver, gut only, and liver only culture: the gut and liver compartment circulation, respectively, and gut-liver interconnection fluid. This device showed its potential for the PK/PD research on the gut-liver axis and extension of a universal multi-organ-on-chip system. In addition to enabling the seeding of specific cells and organs interconnection, flexible modular multi-organ-on-chip can also be integrated with microfluidic or microfabrication devices in a modular manner. Zhang et al. reported an automated modular multi-organ-on-chip platform (Fig. 6b), where the two single organ chips were connected with physical sensors for monitoring extracellular microenvironment parameters, electrochemical biosensors for soluble protein biomarkers detection, bubble trap, and microfluidic breadboard for timed routing of fluids [86]. Long-term monitoring of chronic drug acetaminophen (APAP) using liver–heart chip and short-term evaluation of the acute drug Doxorubicin (DOX) using liver cancer–heart chip were investigated with this system. An instrument with the function of automated culture, perfusion, fluidic coupling, and *in situ* imaging was developed for connecting eight two-channel single-organ chips over a period of three weeks (Fig. 6c) [87]. The bottom of each chip was lined with relevant ECs and was perfused with a universal medium to recapitulate the distribution of the drug and the cross-organ communication. Specific tissues (intestine, liver, kidney, heart, lung, skin, blood–brain barrier and brain) were cultured by perfusing organ-specific medium or exposed to an air-liquid interface in the top channel. This system has successfully translated *in vitro* results into *in vivo* PK parameters (IVIVT) models for nicotine, cisplatin, and drug first-pass metabolism, which were highly similar to the parameters obtained in human clinical studies. A modular four organs-on-chip (heart, liver, bone, skin) reserved the structure features and specific microenvironment of each tissue, while the vascular fluid linked with a universal medium similar to the above study (Fig. 6d) [88]. More importantly, the tissues induced from hiPSCs were cultured with supporting stromal cells in a physiologically relevant ECM [15].

In fact, almost all organs in human body have been studied through organ-on-a-chip technology. Other important organ-on-chips that not discussed in this review include skin [89,90], brain-blood-barrier [91,92], center nervous system [93,94], bone [95,96], eye [97,98], kidney [99,100], etc.

## 3 Biomedical applications

### 3.1 Drug screening

Drug screening is a process that selects the available drug candidates from a large number of lead compounds based on the efficacy, toxicity, and safety, requiring substantial time and financial resources. Even though 2D cell culture and animal models have been regarded as the gold standard in traditional drug development, the lack of systems mimicking complex human physiology hinders the successful extrapolation of *in vitro* results to *in vivo*. To this end, the organ-on-a-chip technology has emerged as a promising platform for drug screening, which integrates physical, chemical and biological cues to addresses the limitations of 2D monolayer testing. Besides, organ-on-chips circumvent ethical issues associated with animal testing. Preclinical drug development involves three main stages. The first stage is the early drug discovery stage, which includes target identification, disease modelling and drug discovery. The second stage is the preclinical screening and testing stage, where the lead optimization and PK/PD studies are conducted. Lastly, the preclinical trial and translational stage focus on drug toxicity and efficacy validation. Organ-on-chip technology enables the involvement of the full process. A wide range of target organ-on-a-chip models have developed to simulate physio/pathophysiological-relevant conditions, and these models are used for certified drugs or preclinical drug testing. Dual/multi-organs-on-chip systems with key organs such as liver, kidney, skin, gut, are constructed for PK/PD studies.

Currently, various disease models are constructed for testing the efficacy of drugs. Classic dual-channels human alveolus-on-a-chip was utilized to analyze the pathological changes and inflammatory responses of distinct cell types to the SARS-CoV-2 [21]. Remdesivir treatment was studied as potential therapeutics, showing the role in inhibition of viral replication and alleviation of epithelial-endothelial barrier disruption. A heart-on-a-chip was fabricated culturing primary neonatal rat cardiomyocytes on the high-speed impedance detection component to evaluate the responses of CMs to drugs [101]. The beating rate and contraction of CMs were observed to be decreased after verapamil treatment, which matched with the approved effect of antiarrhythmic drug. A skin-on-a-chip with the structure of dermis and epidermis was utilized to treat the Curcuma longa leaf extract (CLLE) as efficacy of the cosmetic ingredient. Under the treatment, the skin differentiation and keratinization, and the generation of filaggrin and involucrin were observed, which exhibit its antiaging effects [102]. To accelerate the discovery of anti-cancer drugs, a vascularized tumor-on-a-chip based 96-well plate format was constructed to support tumor spheroid-induced angiogenesis and anti-angiogenic cancer drug screening [103]. The results showed that the addition of 1 mg/mL of bevacizumab and 1.0 µM of sunitinib significantly induced the vascular damage, while the cetuximab was ineffective. For the human physiology, lymphatic vessels drain interstitial fluid from the tissues and also provide a preferential pathway for tumor and anti-tumor drugs *in vivo* [104]. Therefore, it is indispensable to construct a tumor-on-a-chip with the function of blood vessels as well as lymphatic vessels. Ozcelikkale et al. designed a breast cancer chip with the capillary, interstitial, and lymphatic microchannels to better simulate the transportation and absorption of drugs *in vivo* [105]. The response and resistance of breast cancer cell lines (MCF-7 and MDA-MB-231) to doxorubicin were tested on chip, and it was found that the cancer viability was higher than those in 2D models. Another breast cancer-on-a-chip possessing a blood and lymph vessel pair was fabricated by 3D bioprinting technology to investigate the transportation and anti-cancer effect of doxorubicin, with the consideration for different combinations of the blood and lymphatic vessels and tumor cell arrangements [106]. In a study by Ayuso et al., a vascularized tumor-on-a-chip was designed that ECs were coated on the breast cancer cells-embedded collagen hydrogel [107]. NK-92 cells and drugs were perfused from vascular vessels to evaluate the migration through the matrix. It was observed that the immunomodulatory agents such as Programmed Death 1 (PD-1) blockade and Indoleamine2,3-Dioxygenase1 (IDO1) inhibitor can partially alleviate the immune exhaustion of NK-92 cells.

The assessment of drug toxicity is an integral part in the process of drug discovery, leading to more than 60% of drug clinical trials failure [108]. Yu et al. reported a liver-on-a-chip integrated with a heater and active debubbler to study the acute and chronic repeat dosing safety testing using diclofenac and acetaminophen (APAP) [109]. The acute toxicity response caused by APAP was more sensitive in the chip in comparison with static culture, indicating that higher drug concentrations were recommended in 2D cell culture. Another liver-on-chip was constructed using rat, dog, and human hepatic cells for safety and risk assessment of drugs to compare the effects and toxicity of bosentan and fialuridine. Results showed that these drugs exhibited the human-specific effect and toxicity [110]. Furthermore, a blind screening of 27 small molecules on liver-on-chips revealed that 7 out of 8 clinically used drugs exhibited hepatotoxicity. Surprisingly, 50% of these drugs were not detected to be hepatotoxic in the 3D liver sphere models [111]. The findings highlight the superiority of organ-on-a-chip technology over traditional methods for patient safety assessment. Besides, Tavares et al. developed a liver-skin-on-a-chip, which allowed an assessment of toxic or metabolic responses in the liver caused by chemicals capable of penetrating the skin barrier [112]. For other organ-on-a-chip predicting drug-induced toxicity, a heart-on-a-chip was used to culture cardiac microtissues composed of iPSC derived cardiomyocytes, vascular endothelial cells and mural cells to study the isoproterenol-induced cardiac toxicity [113]. The changes of heart rates were recorded through video analysis and calcium imaging. Brown *et al*. constructed a blood–brain barrier (BBB)-on-a-chip to study the BBB permeability to opioids using brain microvascular endothelial cell (BMECs) [114]. To shorten barrier formation time in the device, three changes were applied: differential serum exposure (serum on the vascular side, no serum on the brain side), polyethylene terephthalate, and earlier seeding of astrocytes. The chip properly recapitulated the canonical opioid transport: loperamide, morphine, and oxycodone, respectively. Furthermore, cortisol was found to regulate the opioid transport across the BBB.

Physiology-based PK modeling studies the dynamic changes of drugs over time *in vivo*, such as ADME, using mathematical principles and methods. PD, on the other hand, is used to describe the effect of a drug *in vivo*, including changes of drug efficacy, toxicity, and drug–drug interactions. PK/PD analysis based on accurate organ-on-chip model has more physiological significance compared with traditional compartmental PK model. Tsamandouras et al. developed a polysulfone (PSF)-based gut-liver-on-a-chip to study the PK of diclofenac (DCF) and hydrocortisone (HC), and the gut-liver crosstalk on the on-platform PK-related processes [115]. They found that the interaction between the gut and liver enhanced the intrinsic metabolic clearance of the liver-on-a-chip. A heart-liver-on-a-chip was used to investigate the relationship between temporal PK and PD of terfenadine. The PD effect driven by intracellular drug concentration in cardiomyocytes was weakened in the presence of a metabolically functional liver compartment. A multi-layer organs-on-a-chip, mimicking intestinal wall (absorption), vascular barrier (transportation), hepatic (metabolism), and kidney (excretion), was used to evaluated the PK of ginsenosides compound K (CK) [116]. The distribution and the toxicity of CK in the multi-organ-on-a-chip were compared with 2D culture and single-chip. The studies showed that CK was almost non-toxic to different cells in the organ-on-a-chip because of a lower absorption rate of CK.

### 3.2 Personalized medicine

Although the advancements of organ-on-a-chip technology are accelerating the general drug development, these cannot yet meet the clinical needs because it is unrealistic to extract specific cells from patients, such as cardiomyocyte, neurocyte. Moreover, organ-on-a-chip requires the incorporation of patient's genetic profile reflected in many diseases. The organoid models generated from *in vitro* differentiation culture of human-derived tumors or hiPSCs faithfully retain the key structure and function of the respective organs. Thus, the combination of organoid and organ-on-a-chip technology by taking patient-derived cells or organoids in the precisely controlled microenvironment on-chip can reconstruct the patient-relevant physiology and pathophysiology, accelerating the drug screening, targeted therapeutics assessment in preclinical studies. Notably, the individual drug sensitivity assays using organoids-on-chip helps preventing the unnecessary side effect from patients trial drug [117]. Moreover, organoid-on-chip, as the functional assays model, is more urgent for the precision medicine than genomics since it focuses on the patients’ phenotypic behavior [118].

Conventional organoids culture without physiological microenvironment results in the non-physiological organic characteristics. For instance, intestinal organoids without the fluidic stimulation form the 3D closed cyst-like configurations and lack of lumens [119]. To overcome this limitation, an intestine organoid-on-a-chip was developed by inducing intestinal stem cells to the epithelia with an accessible lumen and mimicking crypt and villus. What's more, the chip incorporated functional perfusable tubes, enabling the colonization of microorganisms for host-microorganism interactions [120]. In this study, the gut organoids were infected with cryptosporidium, resulting in life-threatening diarrhoea. Another research showed that millifluidic flow enhanced vascular kidney maturation, including more mature podocytes and tubular compartments with enhanced cellular polarity and adult gene expression [121]. Furthermore, the application of forskolin under fluidic flow induced the formation of cyst in LTL proximal tubules and distal nephron using kidney organoids from an autosomal recessive polycystic kidney disease (ARPKD) patient's iPSCs [122]. In addition, RAC1 and FOS were identified as two novel therapeutic targets for ARPKD, providing new insight that inhibitors of RAC1 and FOS could be safer for patients of all ages. Archberger et al. developed a retina organoid-on-a-chip that recapitulated the mature photoreceptors with a perfusable vessel network by integrating more than seven kinds of retinal cell types derived from hiPSCs [123]. This chip was able to demonstrated the drug side effects of anti-malaria drug chloroquine and the gentamicin, and the protective effects of the retinal pigment epithelium barrier, showing the promise for drug development and retinal disease modelling.

The personalized medicine based on organoid-on-a-chip is key to clinical cancer treatment because the anti-tumor therapies with severe toxic side effects show variable efficacy in patients [124]. Previously, some patient-derived organoids (PDO) from colorectal and gastroesophageal cancer patients showed high similarity to the patients’ original tumors in phenotypic and genotypic profiling [125]. The response and non-response to targeted agents testing in these PDOs matched the clinical responses in the patients, which shows its great clinical application value. Another case of personalized cancer therapy involves a patient suffering with glioblastoma multiforme (GBM), where the PDO was employed to guide the selection of treatment [126]. Some organoids platforms have combined with high-throughput and high-content technology, showing attractive use for measuring the expression of the genotypes and phenotypes. Maxim et al. reported an organoid platform with targeted RNA-sequencing for screening the drugs effective against colorectal tumors compared with wild-type organoids [127]. In addition, immunotherapy is a therapeutic approach that modulates patients’ immune systems to recognize and attack cancer cells [128]. Due to its safety and efficacy, there is a pressing need for a platform that can evaluate the efficacy of immunotherapy and the combination therapy in preclinical stage [129]. A patients or murine-derived organoid-on-a-chip was used to evaluate the sensitivity and resistance of tumors to immune checkpoint PD-1 blockade [130]. The *in vitro* studies have shown that the TBK1 and CDK4/6 inhibitors can enhance the response to PD-1 blockade. The immunotherapeutic drugs have also been tested to predict the efficacy in individual patients on an organoid-on-a-chip using PDOs from patient-derived head and neck squamous cell carcinoma (HNSCC) [131]. The authors firstly reported the phenomenon of the IDO1 inhibitor-induced immune cells migration towards cancer cells. Jose et al. demonstrated a tumor-on-a-chip to evaluate the exhaustion mechanism of NK cells in a tumor-induced suppressive environment [107]. This suppressive environment can result in functional damage to Natural Killer (NK) cells, which can be improved through the addition of checkpoint inhibitors and immunomodulatory agents.

## 4 Potentials and challenges in organs-on-chips

Organ-on-a-chip technology has experienced explosive development in the past decade. The model overcomes the shortcomings of traditional animal models and *in vivo* models by precisely controlling the microenvironment on chip, showing a huge potential in human organ development study, drug discovery, and diagnosis of human diseases.

The translation and commercialization of organ-on-a-chip technology for pharmacological and medical users face significant challenges related to high-throughput modeling, ease-to-use supporting equipment, and data reproducibility. Currently, PDMS is the most commonly used material in the field of organ-on-a-chip. Although the small-molecule absorption of PDMS is observed in less than 40% of drugs, its application in drug research is still a prevailing concern for researchers [19]. It is widely believed that this absorption is due to the hydrophobicity of PDMS material. Some strategies have been reported to slow down the absorption of highly hydrophobic compounds into PDMS. For example, a PDMS-poly (ethylene glycol) (PEG) block copolymer was incorporated into PDMS to address its inherent hydrophobicity, with a drug pretreatment to eliminate the concentration gradient [132]. This limitation can also be circumvented by the chemical modification on the surfaces of PDMS channel with polydopamine and polynorepinephrine [133], TiO_2_ and glass coatings [134], lipophilic additives [28], etc. Another direct method is to employ the materials that are less absorptive, such as polyurethanes [135], polystyrene [136]. From the manufacturing perspective, the method of PDMS-based soft lithography is regarded as a limitation for industrial development because of the requirement of clean room, low production efficiency, and high-cost [137,138]. As a result, the industrial manufacturing methods like hot pressing and injection molding are receiving increasing attention. Additionally, 3D printing of molds, devices, and even biological tissues offers a solution to the throughput challenge [139]. Compared with conventional photolithography techniques, stereolithography (SLA) and DLP-based 3D printing offer the advantages of complex design prototypes and rapid fabrication. However, the autofluorescence, low toxicity, low optical transparency, and high surface roughness of the 3D-printed pieces prevent their direct use as the cell culture devices. To overcome this, the integration of 3D-printed molds with soft lithography or injection molding allows for fabrication of the PDMS or thermoplastic materials [140,141]. However, the residual oligomers and monomers on the top surface of 3D-printed pieces poison the Pt-based PDMS catalyst, inhibiting the PDMS curing. Various treatments can be employed to address this issue, including a combination UV exposure and heat [142], or the application of a coating [143]. As the primary subtractive manufacturing technology, laser ablation can handle a wide range of materials and allow for automated control of laser motion to create complex geometries. Moreover, laser ablation is a chemical- and contact-free process, as well as the minimal heating and damage to the surrounding area. Notably, laser ablation also enables the use of glass supports/matrices, which is an ideal choice for manufacturing organ-on-a-chip [144]. Additionally, one study showed that nano-membrane with freely chosen geometry could be integrated into a closed-channel system using femtosecond laser [145].

In addition to the materials and manufacturing process, another factor limiting the industry development of the organ-on-a-chip is the manual operation, the low level of automation, and the resulting low throughput and poor reproducibility. The microfluidic technology, such as droplets, micro-wells, and microstructures, can achieve fabricating reproducible and high-throughput 3D cell spheroid with complex cellular architecture [146]. The spheroids can serve as tissue precursors and be cultured on 96/384 format-based organ-on-chips to reproduce the physio/pathophysiological models combined with ECM, stromal cells, and microenvironmental conditions. Furthermore, different tissue precursors can also be cultured in different microcompartments to support multi-organs studies. Regarding the fluid control, devices such as peristaltic pumps, syringe pumps, and diaphragm pumps can provide precise fluid stimulation and nutrient supply to tissues, but they are bulky and do not easily support large-scale tissue culture [147], [148], [149]. Rocker with smaller footprints and lower costs can create the gravity-driven flow by controlling the tilting angle and period, which support high-throughput well plates-based organ-on-a-chip platform [150,151]. Additionally, some robotic fluidic handing units have been developed to aid in cell sampling and fluid control automatically [87,152]. On the other hand, the cumbersome sample size of organ-on-a-chip is not compatible with conventional biochemical detection methods. Open-top and detachable materials/structure designs have been applied to organ-on-a-chip, allowing researchers to remove the tissues or spheroids for a wide range of traditional biomedical assay procedures [153,154]. However, these traditional methods are still labor-intensive and interruptive. Currently, the integrated or modular sensors applied to organ-on-a-chip systems have been developed to achieve real-time sensing of physiological microenvironmental parameters, biomarkers, and more, with high spatial and temporal resolution and diverse information. In addition, artificial intelligence (AI), especially the deep learning (DL) and machine learning (ML), was introduced in the field of organ-on-a-chip to provide comprehensive and convenient data analysis from imaging and kinematic perspective for rapid drug evaluation [155], [156], [157]. The two new strategies combination of the real-time biosensor monitoring integrated on organ-on-a-chip systems and the real-time data analysis by AI is the trend for the next generation of intelligent organ-on-a-chip systems [158].

There are increasing demands for mimicking biological cues in organ-on-a-chip technology, especially the ECM and multicellular architecture. First, the ECM in specific microenvironments has fundamental influence on cell signaling, angiogenesis, and tissue biomechanics, in comparison to normal tissues [159]. In the case of physiological tumor microenvironment, the tumor stroma is composed of a variety of ECM components containing collagen, fibronectin, laminin, proteoglycans, etc. [128] However, the current construction process of tumor organ/organoid-on-a-chip typically involves a single hydrogel or a mixture of two hydrogels. Besides, it is important to note that the ECM in specific ecological niches or physiological state displays various degree of stiffness and hierarchical alignment [160]. For instance, the emphysema leads to a decline in tissue stiffness [161], whereas the fibrotic tissues are characterized by tissue stiffening [162]. Second, another key factor to be considered is that the porous membrane is used to separate different tissues such as epithelia, endothelia, kidney, liver, and the nervous systems to support barrier structure in organ-on-a-chip [163]. The use of supraphysiologically stiff polycarbonate (PC), polyethylene glycol terephthalate (PET), or PDMS-based material is harmful to cell phenotype [159]. Hence, the hydrogel materials with tunable mechanical and chemical properties can promote the physiological relevance of tissue barrier-on-chip [164]. Lastly, the cell-cell interactions are nonnegligible factors for tumor development [165]. The tumor microenvironment contains abundant cell types, including tumor cells, mesenchymal stroma/stem cells (MSCs), ECs, fibroblasts, immune cells, etc. Thus, incorporating vascularization and immunization is crucial for the organ-on-chip model. Complex microphysiological cellular architecture is achievable through the use of primary cell, cell lines, iPSCs from human donors. In contrast to the mixture of different cells, the development of iPSC-driven organoids recreates the self-organizing process during embryogenesis. However, the labor intensive, time-consuming, and inefficient differentiation protocols make it difficult to develop meaningful *in vitro* assays. For the future of iPSCs differentiation, there is a need not only to make use of the standard differentiation schemes currently available from vendors, but also to apply the advanced statistical modeling methods such as design of experiment (DoE) to the development of media formulations that facilitate novel differentiation protocols and reduce cost [166]. Standardized cell production protocols from multiple vendors is also one of the industry priorities for preclinical research innovation in the stem cell field [167]. Besides, the inability to exhibit a fully mature differentiated phenotype and purity of many tissues limit the replication of *in vivo* metabolism and prediction of pharmacology/toxicology [19,168]. Research in the field of organ-on-a-chip highlights the essential role of the microphysiological environment in shaping the structural and functional characteristics of tissues/organs. Consequently, the integration of mechanical, electrical, and fluidic stimuli into iPSCs-driven organoids differentiation protocols has the potential to lead to relatively mature genetic phenotypes in tissues. For instance, iPSCs-driven cardiac tissues were shown the adult-like gene expression and tissue ultrastructure, under the condition of electromechanical stimuli during the period of high cell plasticity and increasing intensity of induced contractions [169]. Additionally, the combination of distinct differentiation protocols and the electrical regulation not only enhances cardiac tissue maturation but also contributes to atrial and *versus* ventricular phenotype divergence [170].

Multi-organ-on-chip systems are a promising technology that aims to replicate the complex and dynamic cross-organ interactions in human body, but it is still far from quantitatively reflecting the human physiology. The cross-organ interactions are established by fluidic connections, which simulate the circulate system to transport nutrients, cell metabolites, etc. Therefore, it is crucial to develop a common medium to support the phenotypes and functions of the organs in these systems. Ideally, all of the soluble specific ligands (hormones, growth factors, cytokines, trace elements, etc.) are required [171], [172], [173]. However, the mixed medium with complementary strategies have been used in the previous models to keep the balance between the multiorgan performance and experimental costs [174]. In addition to the biological considerations, engineering optimization also plays a critical role for the growth of specific multi-organs and exchange of medium. Specific organs can be cultured in the separate chambers, with the interactions being achieved by porous membrane and engineered vessel linking with organ modules [87]. The modular interconnection design also offers a solution to the problem of allometric growth, which refers to the varying growth rates and sizes of different organs. To address this, the respective organ modulars are initiated at different time points that are guided by the scheduled maturation duration and are subjected to an optimally formulated specific tissue medium. Multi-organ-on-chip system is established once the single modular has reached maturation. This system is a simplified version of human organs. Therefore, the “scaling” strategy, considering the effects of relative organ sizes, blood flow rates, cell numbers and ratios of cell types, is critical for replication of human physiology, PK/PD study, and translation from the experiment results on multi-organ-on-chip into the predictions of responses in human body [174]. Various scaling strategies have been purposed in the organ-on-a-chip field. One strategy is “direct scaling”, where the size of the organ is divided by the miniaturization factor to scale down the organs based on human anatomical data [175]. Another popular strategy is allometric scaling, which correlates the mass of organisms with physiological parameters, such as the metabolic rate, the heart rate, and the blood flow rate. According to this strategy, organ size has a nonlinear relationship with the body mass. However, the determination of allometric scaling parameters require extensive experimentation for organ-on-a-chip systems [176]. Alternatively, Wikswo et al. proposed “functional scaling”, which defines the major and quantifiable function of coupled organs [177], such as heart: volume pumped; lung: gas exchanged; liver: metabolism; kidney: molecular filtering and transport; brain: blood-brain barrier function and synapse formation. Functional scaling can allow each organ of multi-organ-on-chip systems to meet the specific functional parameters.

In addition to analyzing the toxicity, efficacy and PK/PD of a wide range of small molecule drugs, organs/organoids-on-chip systems hold great potential for the discovery and development of new modalities as the drug development experience a paradigm shift. Biologics (*e.g.*, monoclonal antibodies (mAbs) and antibody-drug conjugates (ADCs)) are particularly noteworthy due to their high affinity, potency, and particular specificity (low off-target toxicity), making them promising candidates for critical treatments in some serious diseases, such as cancer, cardiovascular and autoimmune diseases [178]. The development process of biologics is closely related to patient needs due to their high species-specificity, which means that the validation results from animal models are deemed less feasible. Moreover, biologics pose a higher risk for unwanted immunogenicity compared to small molecules. It is necessary to predict the immunogenic reaction, its connection to the safety, and PK/PD profiles of this modality, which is limited in 2D models. Nucleic acid-based therapeutics (oligonucleotides), targeting directly RNA or DNA based on sequence complementary as another new modality, continue to gain attention in the development of RNA-based vaccines against SARS-CoV-2 pandemic. Mechanisms of nucleic acid-based therapeutics allow for the great flexibility in their use and personalized medicine. For example, a splice-modulating antisense oligonucleotide drug, specifically tailored to a patient with neuronal ceroid lipofuscinosis 7, assists in reducing the seizures [179]. Hence, as advanced humanized *in vitro* model systems, organoids-on-chip system could greatly facilitate the development of new therapeutics trail and new modality. Besides, this system can simulate and predict drug-induced vascular injury (DIVI) [180], and instability of the oligonucleotide during circulation (degraded by serum nucleases and excreted through the liver and kidneys) [181], which is a recurring challenge in Nucleic acid-based therapeutics development (such as antisense oligonucleotide therapy).

For the validation of organ-on-a-chip model and data, the physiological validation is the initial consideration. Various developed organ-on-chips are assessed by validating the organ/tissue structure and function, as well as conducting certified drug trials to demonstrate the physiological relevance of the models. The physiological scaling aids in the simulating the transition from *in vivo* organs to *in vitro* models. Second, the biological *in vivo* validation depicts the relationship between the *in vitro* data on chip and the *in vivo* responses in the human body. This validation has been accomplished using animal trials. Although organ-on-a-chip technology is intended to replace animal models, in the short term, under the mature drug development models, it can offer the advantages of the reduction in the number of experimental animals, safety assessment of compounds before human trails, and the reduction in the attrition rate of the candidate. Following this, the PK/PD analysis facilitates the prediction of drugs from concentration on the chip to dose in the body. In the third stage, industries validation or regulatory agencies validation, the data of compounds obtained using organ-on-a-chip technology is submitted to the regulatory agencies, where the data is evaluated and classified.

In order to fully realize the benefits and value of organ-on-a-chip technology, multiple stakeholders with multiple disciplines need to be involved, including academia, regulatory agencies, clinical institutions, biotech companies, etc. For instance, during the biopharma drug discovery, significant multifaceted collaboration is required to give a mutual understanding of the overall drug discovery process for evaluation and standardization. In this process, clinical institutions contribute clinical experience and medical expertise, academia contributes organ-on-a-chip technology expertise in biology and engineering, and biologic companies contribute production efficiency and marketing experience. Organ-on-a-chip technology currently is needed a further work to reach the acceptance by the developers and users for drug screening and personalized medicine. Not only is there a need to further improve the quality and quantity, but there is also a need for rigorous standardization of organ-on-a-chip methods to enhance its reproducibility. For example, the control of culture parameters, such as fluid, pH, temperature, lactate, could be standardized. Additionally, the standardization in infrastructure, equipment, medium and biomaterials batches, cell and organ-on-a-chip culture protocol can also enable different users to obtain the results within a certain acceptable margin of error.

## 5 Conclusion

In this paper, we have reviewed recent advances in organ-on-a-chip technology, including lung, gut, heart, liver, vasculature. We also discuss the applications on drug screening and personalized medicine. It attracts worldwide research attention and a large number of organ-on-a-chip have been studied. We believe that organ-on-a-chip technology will be integrated into human-on-a-chip, which plays a vital role in understanding human physiology and pathophysiology, facilitating drug development and personalized medicine.

## Declaration of competing interest

The authors declare that they have no conflicts of interest in this work.
